# Psammoma bodies in a benign thyroid gland: A case report and brief review of the literature

**DOI:** 10.3892/mi.2025.282

**Published:** 2025-10-29

**Authors:** Ari M. Abdullah, Hadeel A. Yasseen, Rawa M. Ali, Shaho F. Ahmed, Abdulwahid M. Salih, Imad J. Habibullah, Twana Omer Saeed, Shko H. Hassan, Harun Amanj Ahmed, Fahmi H. Kakamad

**Affiliations:** 1 Department of Scientific Affairs, Smart Health Tower, Sulaymaniyah 46001, Iraq; 2 Department of Pathology, Sulaymaniyah Teaching Hospital, Sulaymaniyah 46001, Iraq; 3 College of Medicine, University of Sulaimani, Sulaymaniyah 46001, Iraq; 4 Hospital for Treatment of Victims of Chemical Weapons, Halabja 44001, Iraq; 5 Zad Organization, Sulaymaniyah 46001, Iraq; 6 Kscien Organization for Scientific Research (Middle East Office), Sulaymaniyah 46001, Iraq

**Keywords:** psammoma bodies, benign thyroid gland, thyroid calcifications, thyroid follicular nodular disease

## Abstract

Psammoma bodies (PBs) are round, layered calcified structures usually associated with papillary thyroid cancer and may be observed in 40-50% of cases, but may rarely occur in benign thyroid disease. The present study describes a rare case of PBs being found in a benign thyroid gland. In addition, a brief review of the literature is presented. A 28-year-old female patient presented with a 3-month history of weight loss, poor appetite and generalized weakness. Her thyroid was firm and mildly enlarged. Thyroid function was within normal limits (thyroid-stimulating hormone, 0.85 µIU/ml; free T4, 20.1 pmol/l; thyroglobulin, 6.15 ng/ml). An ultrasound demonstrated bilateral TI-RADS-3 nodules measuring 25x21x19 mm (right) and 17x15x13 mm (left). A total thyroidectomy revealed thyroid follicular nodular disease with focal lymphocytic thyroiditis and PBs, but no malignancy. In the literature, 4 cases of PBs or psammomatous calcifications linked to benign conditions were identified, 2 males and 2 females. Half of the cases involved the thyroid-region lesions and half were pediatric (2/4). All cases underwent surgical excision, and fine-needle aspiration was diagnostic in only 1 case (25%). A computed tomography scan was used in 2 cases (50%). A histological analysis confirmed PBs or psammomatous calcifications in every case, and all patients had favorable outcomes with no recurrence upon follow-up. When PBs are detected without tumor cells, submitting the entire thyroid tissue for histology is recommended to rule out microscopic carcinoma. PBs can arise in benign thyroid follicular nodular disease, mimicking malignancy on imaging and cytology, accurate diagnosis requires comprehensive histopathological evaluation.

## Introduction

Psammoma bodies (PBs) are round, layered calcified structures considered to form through dystrophic calcification, a process in which calcium is deposited locally in damaged or dying tissues, despite normal blood calcium levels and no abnormalities in the metabolism of calcium (1). PBs are usually associated with papillary tumors and are most often observed in papillary thyroid carcinoma. Consequently, microcalcifications on an ultrasound are more frequently observed in classic papillary thyroid carcinoma than in other thyroid cancer types, and their histological presence is a hallmark feature of papillary thyroid carcinoma (2). PBs are present in 40-50% of paraffin-embedded sections and in 11-35% of fine-needle aspiration smears of papillary thyroid carcinoma (1). They most often occur on tumor cells located at the tips of papillary structures, likely due to the effects of vascular thrombosis (3). When present in thyroid areas remote from a tumor, PBs are typically found between follicles or within the interlobular septa, regions that contain the lymphatic and vascular structures of the gland (4). However, although rare, PBs can be found in a benign thyroid gland, and their presence without accompanying papillary thyroid carcinoma can pose a diagnostic challenge (5). The present study describes a rare case of PBs found in a benign thyroid gland and also performed a brief review of the literature. The references cited in the present case report have been assessed for reliability (6), and the manuscript was prepared according to the CaReL guidelines (7).

## Case report

### Patient information

A 28-year-old female patient presented to Smart Health Tower (Sulaymaniyah, Iraq) with a 3-month history of weight loss, decreased appetite and generalized weakness, without other associated symptoms. An analysis of her past medical and surgical history did not reveal any notable findings, with no family history of similar conditions and no history of radiation exposure.

### Clinical findings

An examination revealed a firm, mildly enlarged thyroid gland without palpable cervical lymphadenopathy.

### Diagnostic approach

Laboratory investigations revealed that the level of thyroid-stimulating hormone was 0.85 µIU/ml (normal range, 0.8-6.0 µIU/ml) and the level of free T4 was 20.1 pmol/l (normal range, 12.8-27 pmol/l). A neck ultrasound revealed bilateral thyroid nodules, both classified as TI-RADS 3 according to the American College of Radiology TI-RADS system (https://www.acr.org/Clinical-Resources/Clinical-Tools-and-Reference/Reporting-and-Data-Systems/TI-RADS). The right nodule measured 25x21x19 mm, and the left measured 17x15x13 mm. As part of the pre-operative assessment for total thyroidectomy, vocal cord function was evaluated and was normal bilaterally. Additional investigations revealed a thyroglobulin level of 6.15 ng/ml (normal range, 3.5-77 ng/ml) and a serum calcium level of 9.27 mg/dl (normal range, 8.6-10.0 mg/dl).

### Therapeutic intervention

**Figure 1:**
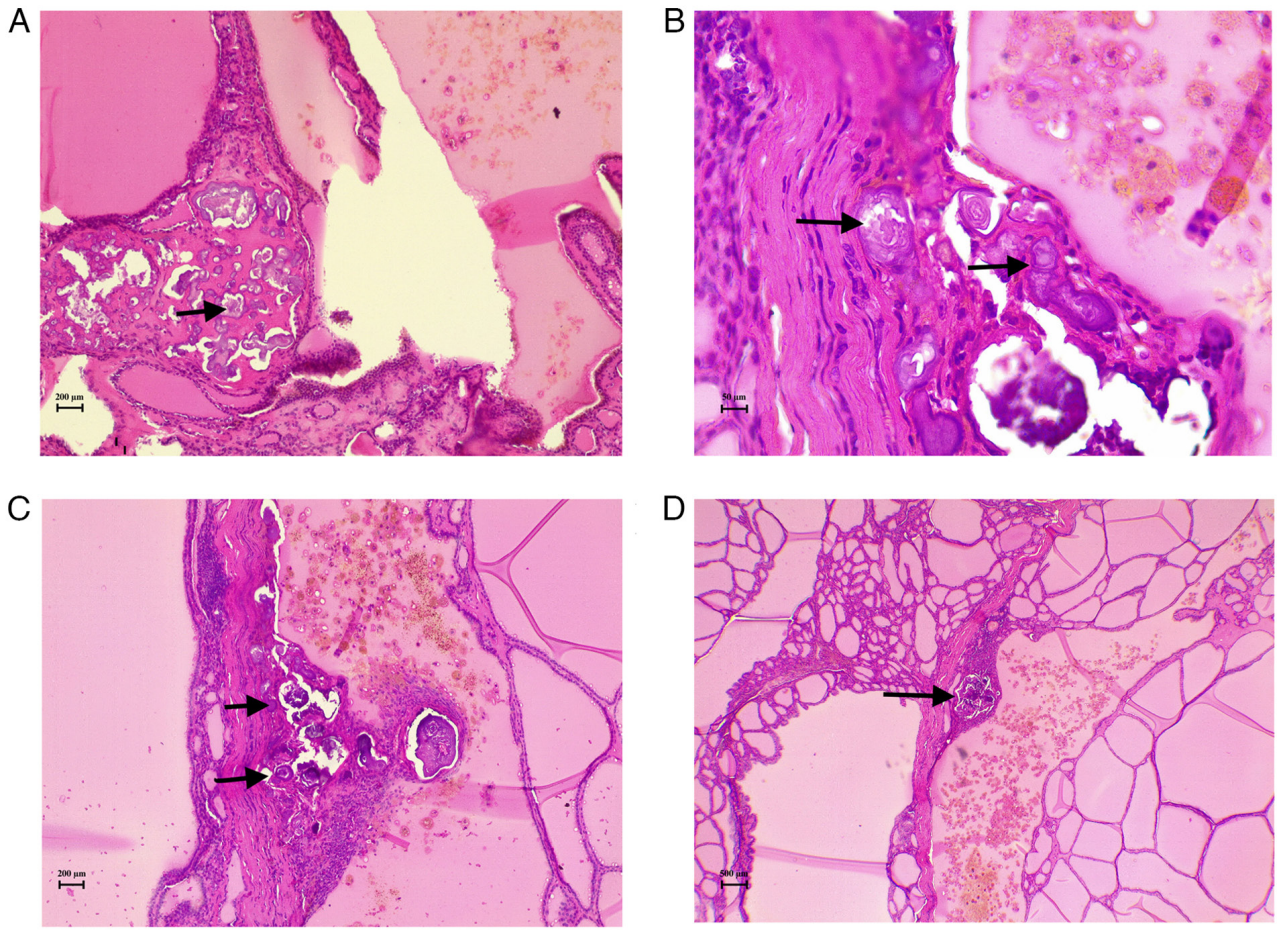
(A-D) Hematoxylin and eosin-stained sections from various areas of the thyroid gland illustrating calcifications, primarily in the form of psammoma bodies (black arrows). Some are surrounded by inflammatory cells, with no associated tumor cells observed. (A and C) magnification, x10; (B) magnification, x40; (D) magnification, x4.

**Figure 2:**
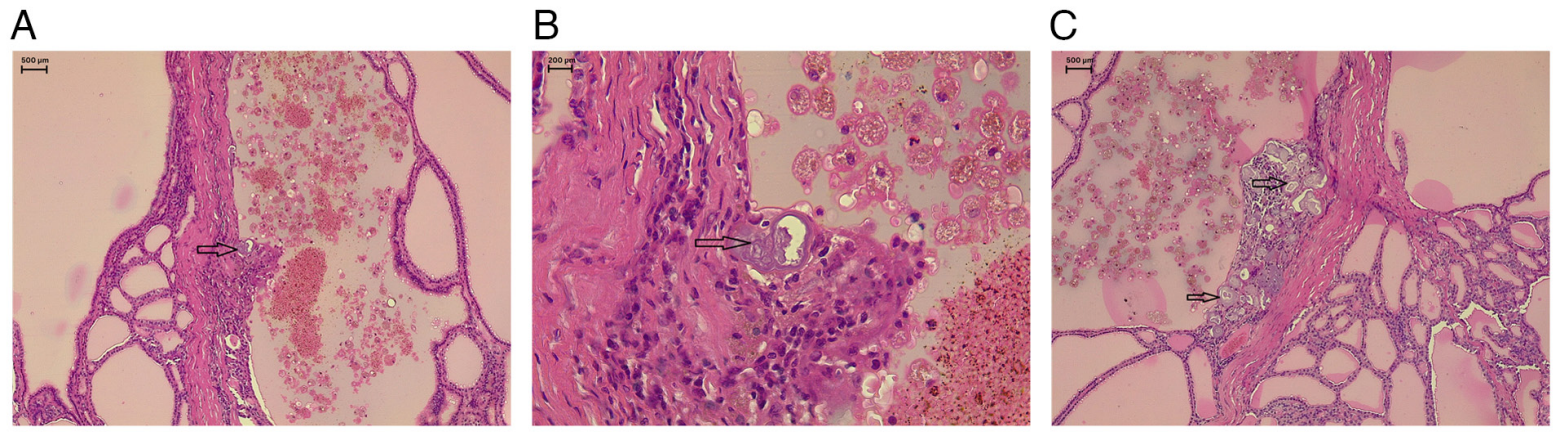
(A-C) Multiple deeper levels from different areas of the thyroid tissue showing the presence of multiple PBs (black arrows) not associated with any suspicious epithelial cell components. Hematoxylin and eosin staining; (A and C) magnification, x4; (B) magnification, x10. PBs are concentrically lamellated (onion-like), round, calcified structures. Histologically, they exhibit a whorled appearance with calcium staining blue, while colloid stains pink and lacks a whorled pattern. By contrast, dystrophic calcifications are typically irregular in shape and lack the concentric lamellations characteristic of PBs. PBs, psammoma bodies.

Under general anesthesia, a total thyroidectomy was performed through a collar incision, with the careful preservation of both recurrent laryngeal nerves and the parathyroid glands. Hemostasis was successfully achieved, and the wound was closed in layers, with a drain placed on the left side. The excised tissue was sent for a histopathological examination. The sections (5-µm-thick) were paraffin-embedded and fixed in 10% neutral buffered formalin at room temperature for 24 h. They were then stained with hematoxylin and eosin (H&E; Bio Optica Co.) for 1-2 min at room temperature. The sections were then examined under a light microscope (Leica Microsystems GmbH). Post-operatively, the patient remained stable with no complications, and serum calcium levels were within the normal range (9.3 mg/dl). The histopathological examination revealed thyroid follicular nodular disease (TFND) with focal lymphocytic thyroiditis, and PBs were identified without any evidence of suspicious epithelial cell components (Figs. 1 and 2). To ensure a comprehensive evaluation, the entire thyroid was submitted for additional sectioning to exclude microscopic carcinoma, no malignancy was identified.

### Follow-up and outcomes

The patient recovered well without complications and was discharged 2 weeks following admission, and was prescribed levothyroxine 100 µg once daily,, with a follow-up appointment scheduled. A multidisciplinary team evaluated her and decided that she would undergo regular follow-ups to monitor for any signs of recurrence.

## Discussion

It is well-established that PBs are commonly associated with carcinomas in different organs, such as papillary thyroid carcinoma, ovarian serous tumors, meningioma and others, and this association is considered diagnostically critical (8). However, PBs may be found in benign conditions, such as TFND with papillary hyperplasia, follicular adenomas, including the oncocytic variant, Hashimoto's thyroiditis and other less common condition (3). A total of 4 cases of PBs and psammomatous calcifications associated with benign conditions were identified in the literature and were reviewed herein; 2 cases were male and 2 cases were female (Table I) (5,9-11). Half of the cases involved thyroid-region lesions and half were pediatric patients (2/4). All 4 cases required surgical excision, while fine-needle aspiration was only used in 1 out of the 4 cases. Computed tomography was used in half the cases (2/4) and other modalities in the remainder. Histopathological analysis confirmed PBs or psammomatous calcifications in every case, and all patients had favorable outcomes with no recurrence documented on follow-up (100%). These findings emphasize that psammomatous calcifications can occur in diverse benign conditions, are best identified by histology, and are commonly managed successfully by surgical resection.

Microcalcifications are observed in <5% of adenomas on thyroid ultrasound, and these correspond to PB clusters when examined cytologically or histologically (12). By contrast, macrocalcifications appear on an ultrasound as bright echogenic spots that produce shallow posterior acoustic shadowing (<1 mm deep), and are less strongly linked to malignancy histologically. Coarse calcifications upon microscopy are irregular dystrophic deposits usually resulting from tissue necrosis. Distinguishing between microcalcifications and macrocalcifications on ultrasound can be challenging, as it depends not only on the level of experience of the operator, but also on the technical capabilities of the imaging equipment (2).

Rossi *et al* (3) evaluated cases of PBs in benign thyroid lesions across five centers over a period of 14 years and identified only 26 patients, with 16 of them being female. The most common diagnoses were TFND and adenomas (12 patients each), of which 11 cases were oncocytic adenomas and 1 case was a follicular adenoma, as well as 2 cases of Hashimoto's thyroiditis. The precise origin and clinical relevance of PBs in non-cancerous conditions are still uncertain and continue to be investigated. The presence of PBs in non-cancerous conditions raises the dilemma of whether PBs in benign lesions should be observed as early signs of malignancy and treated accordingly, or regarded as incidental findings (3).

TFND, which was identified in the current case and was previously referred to as multinodular goiter, is a benign, non-inflammatory condition marked by an enlarged thyroid gland and multifocal proliferation of thyroid follicular cells, resulting in clonal and non-clonal nodules with diverse architectures. It is more frequently observed in women, and iodine deficiency resulting in an elevated thyroid-stimulating hormone release, which stimulates hyperplasia of thyroid follicular epithelial cells, is considered a primary contributing factor. TFND may present with diverse histological features, that mimic well-differentiated thyroid carcinomas. Distinguishing between concerning histological changes resulting from a benign hyperplastic process and those indicative of true neoplastic transformation is essential (13).

Ultrastructural studies of PBs of the thyroid found in both tumor and non-tumor areas, suggests that they likely represent the final stage of two biological processes: The first involves the basement membrane in the vascular core of neoplastic papillae becoming thickened, followed by vascular thrombosis, calcification and tumor cell death. Secondly, necrosis and calcification occur within the intralymphatic tumor thrombi, which are located either near the tumor or in the opposite lobe of the thyroid (1). PBs should be differentiated from calcified colloid and dystrophic calcification (4). There is an increased presence of PBs and a higher frequency of lymph node metastases in tumors with rearranged during transfection (RET) rearrangements. This indicates a potential complex association between RET pathway alterations, PB formation and nodal involvement (14). PBs located outside the tumor in patients with classic type papillary thyroid carcinoma may be linked to a greater likelihood of multifocal tumors, spread beyond the thyroid, and lymph node metastasis compared to tumors that contain PBs only within the tumor itself. Non-tumor PBs in patients with lymph node involvement also exhibit a notably increased rate of viable tumor cells present within lymphatic vessels compared to those without such findings (14).

For the histopathological analysis of PBs, the tissue is fixed in 10% buffered formaldehyde, embedded in paraffin, sectioned into 5-µm-thick slices, and subsequently stained with hematoxylin and eosin (3). PBs are frequently observed to be encircled by cells and typically display a concentric pattern, appearing either acidophilic or basophilic when stained with Papanicolaou stain (8). PBs are less commonly detected in fine-needle aspiration cytology, although their presence strongly indicates carcinoma. However, they are not dependable indicators unless accompanied by other distinct cytological features such as nuclear grooves, nuclear pseudoinclusions and true papillary formations (12). Ellison *et al* (15) reported that PBs were identified in only eight of 313 fine-needle aspirates, PBs alone had a 50% positive predictive value for papillary carcinoma, whereas combined cytological features were 100% predictive. PBs were found in 80% of papillary thyroid cases when combined features were present compared with 14% detection of PB alone in their study (15).

The presence of PBs in benign lesions remains a topic of diagnostic uncertainty. Submitting the entire thyroid tissue for histological analysis, particularly in lobectomy specimens, is advised to rule out microscopic papillary thyroid carcinoma (4). Multiple and deeper sections of thyroid parenchyma need to be examined to search for occult carcinoma. However, some cases remain tumor-free, supporting the occurrence of PBs in benign conditions (3). Hunt and Barnes (4) evaluated 29 patients (22 females) with PBs not initially associated with tumors. A histological examination, however, revealed carcinoma in the thyroid gland in 27 patients, and 12 of these were observed alongside microscopic, incidental thyroid carcinomas, emphasizing the importance of thoroughly sampling the entire surgical specimen. In their study, 23 patients had PBs associated with high-risk features according to the AMES criteria, as well as the presence of the tall cell variant as an added risk factor (4). A total of 5 patients experienced disease recurrence, but no deaths were attributed to thyroid cancer. In the total of 29 patients, a total of 19 patients had a total thyroidectomy, while ten others underwent lobectomy. The 2 patients without any carcinoma underwent a lobectomy, and the entire surgical specimen was not sent for a histopathological examination (4). In the study by Rossi *et al* (3), 24 out of the 26 patients with PBs underwent a total thyroidectomy.

Since the patient in the present study underwent a simple total thyroidectomy and PBs were identified afterward, intraoperative neuroendoscopy and magnetic resonance imaging were not performed. It should be noted that sonographic categorization is subject to both operator expertise and equipment variability, which may influence nodule classification and reproducibility, including this case.

In conclusion, PBs may occur in benign TFND and can mimic malignancy on imaging and cytology, definitive diagnosis relies on thorough histopathological sampling.

## Figures and Tables

**Table I:** Review of some cases of PBs and psammomatous calcifications associated with benign conditions in the literature.

First author, year of publication	Age, years	Sex	Diagnosis	Clinical findings	Imaging tests	Microscopic examinations	Fine needle aspiration	Treatment	Outcome	(Refs.)
Cardisciani, 2022	74	F	Pseudonodular hyperplastic oncocytic metaplasia in a background of chronic lymphocytic Hashimoto thyroiditis.	History of multinodular goiter pressing on the trachea.	Not mentioned.	Lymphocytic Hashimoto thyroiditis with nodular oncocytic metaplasia, and PBs. IHC: -ve HBME-1, galectin-3 and CK-19.	Not performed.	Total thyroidectomy.	Is in stable condition.	(5)
Mohammed, 2015	9	M	Leiomyoma of the thyroid gland.	Painless thyroid mass.	X-ray: Tracheal deviation to the left.	PBs and foci of calcification. HPE: +ve SMA, vimentin and desmin, -ve cytokeratin cocktail.	Benign smooth muscle tumor.	Right hemi-thyroidectomy.	No recurrence after 2 years.	(9)
Vemavarapu, 2011	46	F	Benign Brenner tumor of the ovary.	Progressively enlarging pelvic mass.	US: Cystic lesion in the left adnexa with punctate calcifications. CT: solid components and calcifications.	Dystrophic calcifications lining the cyst wall, obscuring the epithelium, largely replacing it, with extensive stromal PC, and no nuclear atypia or mitosis.	Not mentioned.	Total robotic hysterectomy and bilateral salpingo-oophorectomy.	Is in stable condition.	(10)
Ayala, 2003	3	M	Benign thyroglossal duct cyst.	Asthma symptoms.	CT: Hyoid bone mass.	Thyroglossal duct remnant with cystic spaces, and PC, no evidents of malignancy.	Not mentioned.	Excision of the mass.	Is in stable condition.	(11)

M, male; F, female; IHC, immunohistochemistry; +ve, positive; -ve, negative; PBs, psammoma bodies; PC, psammomatoid calcifications; SMA, smooth muscle actin; TTF-1, thyroid transcription factor-1; US, ultrasound; CT, computed tomography; MRI, magnetic resonance imaging.

## Data Availability

The data generated in the present study may be requested from the corresponding author.
